# Infrared Stealth Characteristics of WO_3_-Based Electrochromic Devices Mediated by Zn^2+^-Al^3+^ Gel Electrolyte

**DOI:** 10.3390/ma19081506

**Published:** 2026-04-09

**Authors:** Ke Wang, Xiaoting Yang, Tongyu Liu, Wei Zhang

**Affiliations:** 1 School of Microelectronics, Tianjin University, Tianjin 300072, China; keywang2020@163.com; 2 National Key Laboratory of Electromagnetic Space Security, Tianjin 300308, China; 3 China Electronics Technology Group Corporation, Optoelectronics Research Institute, Tianjin 300308, China

**Keywords:** tungsten oxide, electrochromic device, gel electrolyte, zinc-ion doping, infrared stealth, emissivity modulation

## Abstract

As one of the core technologies in modern national defense and security fields, infrared stealth technology aims to realize the controllable regulation of the radiation characteristics of targets in the infrared band. This paper focuses on a novel electrochromic device with a structure of WO_3_/nickel mesh/Al^3+^-Zn^2+^gel electrolyte/zinc foil. The structural composition and working mechanism are systematically analyzed, and the infrared stealth regulation performance is emphatically studied. The WO_3_ thin film and device structure were characterized by scanning electron microscopy (SEM). The infrared emissivity modulation and optical response properties of the device were measured using an infrared thermal imager and a UV-Vis-NIR spectrophotometer. The prepared WO_3_ film exhibits a dense spherical morphology, indicating excellent uniformity and compactness. After 1000 cycles, the areal capacitance of the device remains 83.7% of its initial value, demonstrating good cycling stability. Under the voltage regulation of −0.1 V to 1.1 V, the emissivity ε of the device at the typical mid-wave infrared wavelength of 4.0 μm decreases from 0.89 (−0.1 V) to 0.67 (1.1 V), with an absolute modulation amplitude Δε of 0.22. At the typical long-wave infrared wavelength of 8.7 μm, ε decreases from 0.96 (−0.1 V) to 0.69 (1.1 V), with an absolute modulation amplitude Δε of 0.29. The electrochromic switching times for coloring and bleaching are 10.1 s and 2.44 s, respectively. According to infrared thermal imaging tests, in the temperature range of 30–40 °C, the surface temperature difference ΔT between the colored state and bleached state increases from 4.3 °C to 4.6 °C. The maximum regulation amplitude reaches 4.6 °C at 40 °C. The device achieves efficient regulation of infrared emissivity through the electrochromic effect, providing a new device design strategy for infrared stealth technology.

## 1. Introduction

As one of the core technologies in modern national defense and aerospace fields, infrared stealth technology aims to reduce the infrared radiation contrast between targets and backgrounds by regulating the infrared emissivity of targets, thereby avoiding detection by infrared detection systems. Electrochromic materials have become ideal candidates for fabricating smart infrared stealth devices due to their ability to reversibly modulate optical properties (including those in the infrared band) under an applied electric field. Among them, tungsten trioxide (WO_3_), as a typical cathodic electrochromic material, has been widely studied in infrared stealth devices owing to its wide infrared emissivity modulation range, good chemical stability, and mature preparation process [1,2,3,4].

As a key component of electrochromic devices, the ionic conductivity, stability, and compatibility with electrode materials of the electrolyte directly affect the response speed and cycle life of the device. The electrolytes commonly used in electrochromic devices mainly include gel electrolytes, liquid electrolytes, and solid electrolytes. Liquid electrolytes exhibit high ionic conductivity and fast response speed, but suffer from problems such as easy leakage, volatilization, and poor compatibility with electrodes, which tend to cause device performance degradation during long-term operation. Although solid electrolytes solve the leakage issue, their low ionic conductivity and large interfacial resistance limit the coloring/bleaching efficiency and cycling stability of the device. At present, optimizing the encapsulation and compatibility of liquid electrolytes and improving the ion transport efficiency of solid electrolytes remain key research directions for electrolyte systems in electrochromic devices. Gel electrolytes combine the advantages of the high ionic conductivity of liquid electrolytes and good mechanical stability of solid electrolytes, effectively solving the problem of easy leakage of liquid electrolytes. Aluminum ions have a small ionic radius and fast migration rate, and are commonly used as dopant ions for gel electrolytes. However, gel electrolytes doped with single aluminum ions suffer from limited ionic conduction efficiency. Zinc ions have an ionic radius similar to that of aluminum ions, and Zn^2+^ exhibits a high charge density, which can produce a synergistic effect with aluminum ions to further improve the ionic conductivity of the electrolyte. In addition, the electrode structure of the device also affects the efficiency of ion transport and electron conduction. Nickel mesh has good electrical conductivity and a porous structure, which can be used as a supporting substrate for WO_3_ thin films and improve the mechanical strength of the device [5,6,7,8,9,10,11,12,13].

Based on the above considerations, this work designed an electrochromic device with a structure of WO_3_/nickel mesh/Zn^2+^-doped Al^3+^ gel electrolyte/zinc foil. A WO_3_ thin film deposited on nickel mesh was used as the working electrode, zinc foil as the counter electrode, and Zn^2+^-doped Al^3+^ gel as the electrolyte. The microstructure, electrochemical performance, and infrared stealth regulation performance of the device were systematically investigated, aiming to develop a high-performance smart infrared stealth device.

## 2. Materials and Methods

### 2.1. Device Fabrication

#### 2.1.1. Pretreatment of Nickel Mesh

Nickel mesh (pore size 25.4 mm, thickness 0.1 mm) was cut into a size of 2 cm × 3 cm. It was ultrasonically cleaned with acetone, ethanol, and deionized water for 15 min in sequence to remove surface oil and impurities, and then dried in a vacuum drying oven at 60 °C for 2 h for later use.

#### 2.1.2. Fabrication of WO_3_ Thin Films

WO_3_ thin films were deposited on the pretreated nickel mesh substrate by direct current (DC) reactive magnetron sputtering. The optimized process parameters are as follows: The nickel mesh substrate was fixed on the sample stage in the sputtering chamber, and a metallic tungsten target with a purity of 99.95% was used as the sputtering source. The chamber was first evacuated until the vacuum level reached above 5 × 10^−4^ Pa. Then, argon (Ar) and oxygen (O_2_) were introduced as working gases, the volume ratio of O_2_ to Ar was adjusted to 1:1, and the working pressure was stabilized at 1.0 Pa. The sputtering power was set to 5 W/cm^2^, and pre-sputtering was performed for 5 min to remove the oxide layer and impurities on the tungsten target surface, followed by formal sputtering for 60 min. After sputtering, to improve the crystallization quality of the WO_3_ thin films, the sample was annealed in an atmospheric environment at a heating rate of 100 °C/h, kept at 450 °C for 2 h, and cooled naturally to obtain the WO_3_/nickel mesh working electrode.

#### 2.1.3. Preparation of Zn^2+^-Doped Al^3+^ Gel Electrolyte

PEG-6000 (10 g) was dissolved in 50 mL deionized water and magnetically stirred at 80 °C until fully dissolved to obtain an aqueous PEG solution. AlCl_3_·6H_2_O (0.2 mol/L) and ZnCl_2_ (0.05 mol/L) were added separately, and stirring was continued for 1 h. The mixed solution was then poured into a Petri dish and dried in a vacuum drying oven at 60 °C for 12 h to remove water, yielding the Zn^2+^-doped Al^3+^ gel electrolyte.

#### 2.1.4. Preparation of Electrochromic Device

The electrochromic device was assembled using WO_3_/nickel mesh as the working electrode and zinc foil (2 cm × 3 cm × 0.2 mm) as the counter electrode. The as-prepared Zn^2+^-doped Al^3+^ gel electrolyte was uniformly coated between the two electrodes to ensure full coverage of the effective electrode area. The assembly was then kept at room temperature for 2 h to allow the electrolyte to solidify, completing the fabrication of the WO_3_/nickel mesh/Zn^2+^-doped Al^3+^ gel electrolyte/zinc foil electrochromic device.

### 2.2. Characterization and Performance Measurements

#### 2.2.1. Microstructural Characterization

Scanning electron microscopy (SEM) was used to observe the morphology, size, and elemental distribution of the nickel mesh and WO_3_ thin films.

#### 2.2.2. Electrochemical Characterization

Cyclic voltammetry (CV) curves of the device were recorded using an electrochemical workstation. The voltage range of the device was −0.1 V to 1.1 V, with a scan rate of 50 mV/s, and the voltage range of the device electrode was −0.8 V to 0.6 V, with a scan rate of 50 mV/s. The difference in the voltage ranges originates from the different test configurations: the device was tested in a two-electrode system, while the individual electrode was characterized using a three-electrode configuration.

#### 2.2.3. Infrared Stealth Performance Measurement

An infrared thermal imager was used to record the surface temperature distribution of the device at different voltages, with the ambient temperature controlled at 25 °C.

The infrared emissivity of the device in the 2.5–25 μm band was measured using a UV-Vis-NIR spectrophotometer, Shimadzu, Kyoto, Japan.

#### 2.2.4. Cycling Stability Measurement

The device was cycled between −0.1 V and 1.1 V for 1000 cycles to evaluate its long-term stability. The scanning voltage range determines the operating voltage of the electrochromic electrode. The reasonable selection of the scanning voltage range must meet two requirements: the upper voltage limit should be lower than the decomposition potential of the electrolyte to avoid side reactions, and the lower voltage limit should ensure the sufficient progress of the redox reactions of the electrode material.

## 3. Results and Discussion

### 3.1. Microstructural Characterization of the WO_3_/Nickel Mesh Electrode

**Figure 1:**
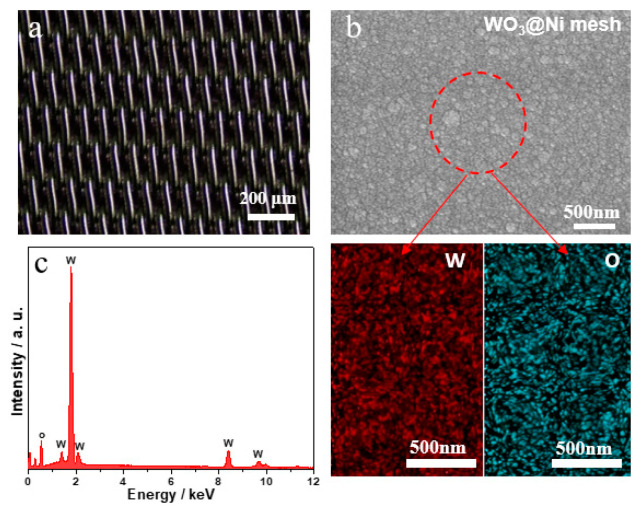
(**a**) SEM image of the nickel mesh surface; (**b**) SEM image and elemental mapping of the WO_3_/Ni mesh electrode; (**c**) EDS spectrum of the WO_3_/Ni mesh electrode.

As shown in Figure 1a, the Ni mesh substrate exhibits a network structure, which can increase the contact area between the WO_3_ thin film and the Ni mesh substrate, thereby improving the adhesion between the film and the substrate. Meanwhile, the network structure of the Ni mesh substrate is also beneficial to the ion transport of the gel electrolyte. Figure 1b shows the SEM image of the WO_3_/Ni mesh electrochromic electrode. It can be seen that the WO_3_ thin film presents a dense spherical structure, indicating excellent uniformity and compactness. Elemental mapping of the selected area confirms that W and O elements are uniformly distributed on the film surface. Figure 1c shows the energy-dispersive X-ray spectroscopy (EDS) pattern of the WO_3_/Ni mesh electrode, which further verifies the existence of W and O elements on the electrode surface.

### 3.2. Electrochemical Performance Analysis of the WO_3_/Nickel Mesh Electrode

**Figure 2:**
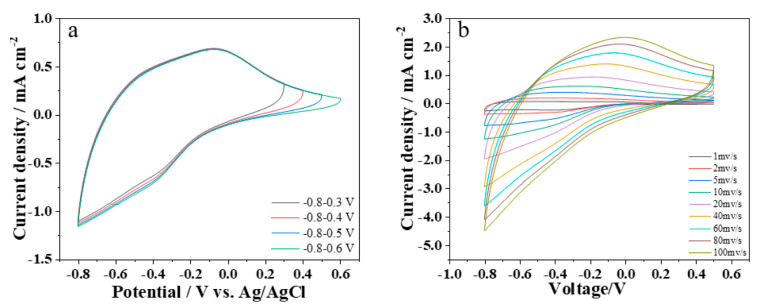
(**a**) CV curves of the WO_3_/Ni mesh electrochromic electrode at different voltage ranges; (**b**) CV curves of the WO_3_/Ni mesh electrochromic electrode at different scan rates.

The electrochemical performance of the WO_3_/Ni mesh electrode was investigated using a three-electrode system with a Zn^2+^ solution as the electrolyte, Ag/AgCl as the reference electrode, and a platinum sheet as the counter electrode. The scanning voltage range determines the operating voltage of the electrochromic electrode. The reasonable selection of the scanning voltage range must meet two requirements: the upper voltage limit should be lower than the decomposition potential of the electrolyte to avoid side reactions, and the lower voltage limit should ensure the sufficient progress of the redox reactions of the electrode material. Figure 2a shows the CV curves of the WO_3_/Ni mesh electrochromic electrode in different voltage ranges. It can be seen that the WO_3_/Ni mesh electrode exhibits a large envelope area in the voltage range of −0.8 V to 0.6 V. Therefore, the voltage range for the electrochemical test of the WO_3_/Ni mesh electrochromic electrode was determined as −0.8 V to 0.6 V. As shown in Figure 2b, the WO_3_/Ni mesh electrochromic electrode displays typical CV curves at scan rates from 1 to 100 mV/s, with distinct redox peaks. This fully demonstrates that continuous redox reactions can occur in the WO_3_/Ni mesh electrode within the scanning voltage range of −0.8 V to 0.6 V. The corresponding electrochromic reaction of WO_3_ is:

WO_3_ + xM^+^ + xe^−^ ↔ MₓWO_3_ (M^+^ = Al^3+^, Zn^2+^) (1)

The reduction peak (coloration process) is located at approximately −0.8 V, corresponding to the intercalation of M^+^ into the WO_3_ lattice. The oxidation peak (bleaching process) is located at about 0.6 V, corresponding to the extraction of M^+^ from the WO_3_ lattice [14,15,16,17,18,19,20].

### 3.3. Electrochemical Performance of the WO_3_/Ni Mesh/Zn^2+^-Al^3+^/Zn Foil Electrochromic Device

**Figure 3:**
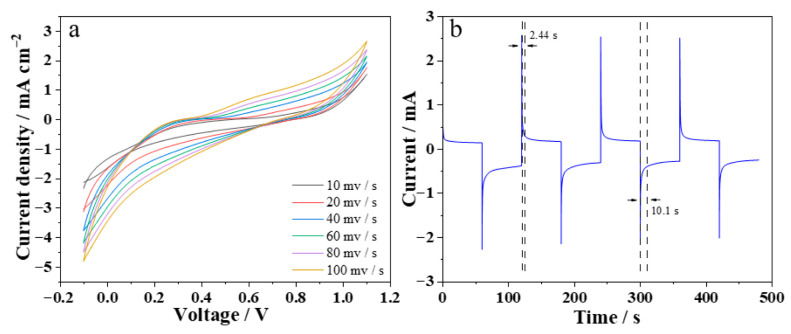
Electrochemical performance of the WO_3_/Ni mesh electrochromic device: (**a**) CV curves at different scan rates; (**b**) response time.

The electrochemical performance of the device was characterized by cyclic voltammetry. Figure 3a shows the CV curves of the electrochromic device at different scan rates. It can be observed that the potential difference between the oxidation peak and the reduction peak gradually increases with increasing scan rate, which is caused by the increased ion diffusion resistance within the film. As can be seen from Figure 3b, the bleaching time and coloration time of the device are 2.44 s and 10.1 s, respectively, indicating a fast response speed for switching between the high-emissivity state and the low-emissivity state. This endows the device with potential application value in the field of high-sensitivity and high-dynamic infrared stealth.

**Figure 4:**
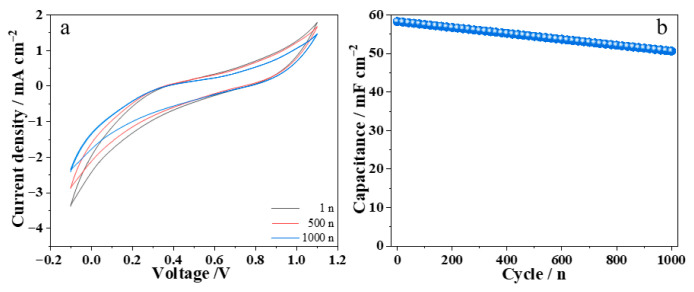
Cyclic stability test of WO_3_/Ni mesh electrochromic device.

Cycling stability is another important parameter for evaluating the performance of electrochromic devices. Figure 4 shows the cycling stability test results of the WO_3_/Ni mesh electrochromic device. As can be seen from Figure 4a, after 1000 voltage cycles, the CV curve of the device still maintains high consistency with the initial curve. As shown in Figure 4b, after 1000 cycles, the areal capacitance of the device electrode remains 83.7% of its initial value. This fully demonstrates that the WO_3_/Ni mesh/Zn^2+^-Al^3+^/Zn foil electrochromic device exhibits high reliability and excellent stability [15,16,17,18,19,20,21,22,23,24].

**Figure 5:**
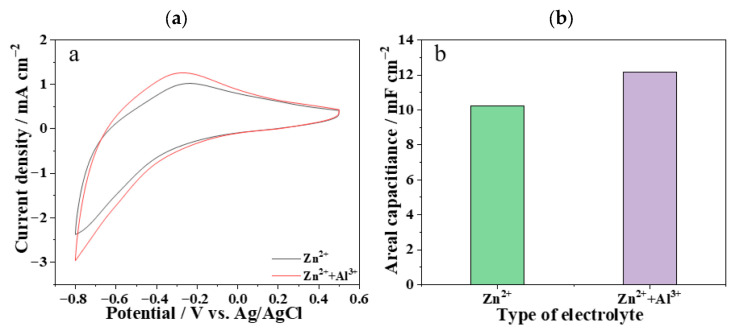
(**a**) CV curves of WO_3_/Ni mesh electrochromic electrode in different electrolytes; (**b**) areal capacitance of the device electrode under different electrolytes.

As shown in Figure 5, compared with the electrolyte containing only Zn^2+^, the doping of Al^3+^ in the electrolyte increases the areal capacitance of the electrochromic device electrode, which further indicates that the synergistic effect of Zn^2+^ and Al^3+^ improves the cycling stability of the device.

### 3.4. Infrared Modulation Performance of the WO_3_/Ni Mesh/Zn^2+^-Al^3+^/Zn Foil Electrochromic Device

**Figure 6:**
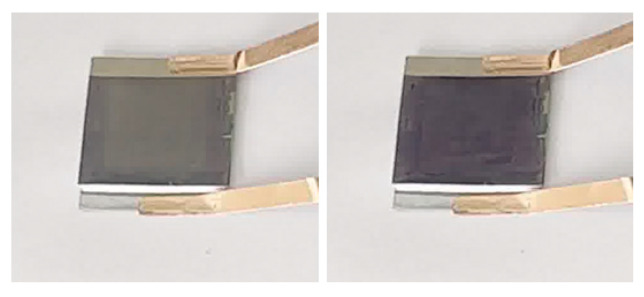
Optical photograph of the WO_3_/Ni mesh electrochromic device.

Figure 6 shows the optical image of the WO_3_/Ni mesh/Zn^2+^-Al^3+^/Zn foil electrochromic device.

**Figure 7:**
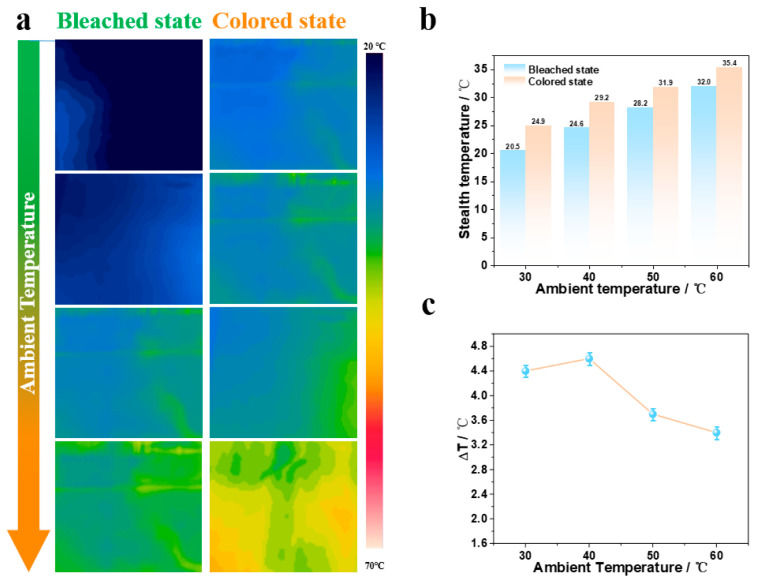
Infrared modulation performance of the WO_3_/Ni mesh electrochromic device. (**a**) Infrared thermograms of the colored and bleached states at different background temperatures; (**b**) radiation temperatures of the colored and bleached states at different background temperatures; (**c**) radiation temperature adjustment ranges of the colored and bleached states at different background temperatures.

Figure 7 shows the infrared thermal imaging test results of the device, characterizing the dynamic regulation performance of the WO_3_/Ni mesh/Zn^2+^+Al^3+^/Zn foil electrochromic device on infrared radiation at different ambient temperatures. Figure 7a shows the surface infrared radiation temperatures of the device in the colored state (−0.1 V) and bleached state (1.1 V) under different ambient temperatures. As shown in Figure 7b, when the ambient temperature increases from 30 °C to 60 °C, the surface radiation temperature of the device in the colored state rises from 24.9 °C to 35.4 °C, while the surface temperature in the bleached state increases from 20.5 °C to 32 °C. Figure 7c displays the variation trend of the radiation temperature difference ΔT (T_colored-T_bleached) of the device at different ambient temperatures. In the temperature range of 30–40 °C, ΔT increases from 4.4 °C to 4.6 °C. When the temperature exceeds 40 °C, ΔT begins to decrease to 3.4 °C at an ambient temperature of 60 °C. The device reaches the maximum modulation amplitude of 4.6 °C at 40 °C. The results indicate that the device possesses infrared radiation modulation capability at different ambient temperatures, which provides a promising potential for the application of the device in the field of infrared stealth.

**Figure 8:**
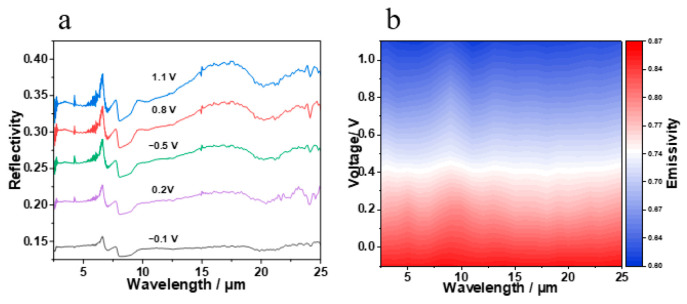
(**a**) Infrared reflectance spectra of the electrochromic device at different voltages; (**b**) contour plot of spectral emissivity.

Infrared reflection spectra (2.5–25 μm) of the device at different driving voltages were measured using a Fourier transform infrared (FT-IR) spectrometer with a wavelength range of 2.5–25 μm. The results are shown in Figure 8a. It can be seen that when the voltage is scanned from −0.1 V (colored state) to 1.1 V (bleached state), the reflectivity of the device in the 2.5–25 μm band shows an upward trend. According to Kirchhoff’s law of thermal radiation (ε(λ) = 1 − R(λ)), at the typical mid-wave infrared wavelength of 4.0 μm, the emissivity ε decreases from 0.89 (−0.1 V) to 0.67 (1.1 V), with an absolute modulation amplitude Δε of 0.22. At the typical long-wave infrared wavelength of 8.7 μm, ε decreases from 0.96 (−0.1 V) to 0.69 (1.1 V), with an absolute modulation amplitude Δε of 0.29. This indicates that the device exhibits dynamic infrared modulation performance in both the mid-wave and long-wave infrared bands.

Based on the formula

ε = 1 − R(2)

emissivity contour curves of the electrochromic device in the wavelength range of 2.5–25 μm were plotted (Figure 8b). As the applied voltage increases, ε gradually decreases, demonstrating that the electrochromic device possesses dynamic infrared emissivity modulation capability in the infrared region.

### 3.5. Analysis of Modulation Mechanism

(1) Ion Synergistic Regulation Mechanism

The Zn-Al composite gel electrolyte provides synergistic transport pathways for Zn^2+^/Al^3+^. Owing to their high mobility and strong lattice distortion effect, respectively, the two ions cooperatively intercalate into and deintercalate from the WO_3_ lattice, which significantly broadens the emissivity modulation range in the dual infrared atmospheric windows of 3–5 μm and 8–14 μm, realizing dynamic control of infrared radiation.

(2) Interface and Stability Enhancement Mechanism

As a conductive substrate for WO_3_, the nickel mesh improves electron transport efficiency and film adhesion, ensuring the synergistic transmission of ions and electrons. The three-dimensional network of the Zn^2+^-Al^3+^ gel confines ion migration, suppresses Zn^2+^ dendrite growth and interfacial side reactions, optimizes ionic conductivity, shortens the infrared response time, and enhances the cycling stability of the device.

(3) Ion–Electron Matching Transport Mechanism of the Whole Structure

The zinc foil anode, nickel mesh conductive substrate, WO_3_ electrochromic layer, and Zn^2+^-Al^3+^ gel electrolyte form an efficient ion–electron transport circuit, which guarantees the synchronism and uniformity of dual-ion intercalation/deintercalation. This avoids the fluctuation of infrared emissivity caused by uneven local lattice distortion, and improves the uniformity and consistency of infrared modulation [25,26,27,28,29,30].

## 4. Conclusions

In this study, an electrochromic device with a structure of WO_3_/nickel mesh/Zn^2+^-doped Al^3+^ gel electrolyte/zinc foil was successfully fabricated. Its microstructure, electrochemical properties, and infrared stealth modulation performance were systematically investigated, and the conclusions are as follows:

(1) The as-prepared WO_3_ thin film exhibited a dense spherical morphology, indicating excellent uniformity and compactness. The nickel mesh substrate improved the conductivity and mechanical strength of the film. The device showed good cycling stability: after 1000 cycles, the areal capacitance of the electrode remained 83.7% of its initial value. The doping of Al^3+^ in the electrolyte enhanced the areal capacitance of the electrochromic electrode, further demonstrating that the synergistic effect between Zn^2+^ and Al^3+^ improved the cycling stability of the device.
(2) Under voltage modulation between −0.1 V and 1.1 V, at a typical mid-wave infrared wavelength of 4.0 μm, the emissivity ε decreased from 0.89 (−0.1 V) to 0.67 (1.1 V), with an absolute modulation amplitude Δε of 0.22. At a typical long-wave infrared wavelength of 8.7 μm, ε decreased from 0.96 (−0.1 V) to 0.69 (1.1 V), with an absolute modulation amplitude Δε of 0.29. The coloring/bleaching response times were 10.1 s and 2.44 s, respectively, revealing fast and efficient infrared stealth modulation capability.
(3) In the temperature range of 30–40 °C, the surface temperature difference ΔT between the colored state and the bleached state increased from 4.4 °C to 4.6 °C. When the temperature exceeded 40 °C, ΔT gradually decreased to 3.4 °C at an ambient temperature of 60 °C. The device reached the maximum modulation amplitude of 4.6 °C at 40 °C. These results confirm that the device possesses effective infrared radiation modulation ability at various ambient temperatures, which provides promising application potential in the field of infrared stealth.

## Data Availability

The original contributions presented in this study are included in the article. Further inquiries can be directed to the corresponding authors.
